# Metastatic non-muscle invasive bladder cancer with meningeal carcinomatosis: case report of an unexpected response

**DOI:** 10.1186/s12885-017-3309-2

**Published:** 2017-05-11

**Authors:** Diego Teyssonneau, Amaury Daste, Vincent Dousset, Jean-Luc Hoepffner, Alain Ravaud, Marine Gross-Goupil

**Affiliations:** 1 Department of Medical Oncology, Saint-André hospital, University Hospital-CHU Bordeaux, 1 Rue Jean Burguet, 33000 Bordeaux, France; 2 University of Bordeaux, Bordeaux, France; 3 Neuroradiology Department, Bordeaux University Hospital, CHU Bordeaux, 33000 Bordeaux, France; 4 Department of Urology, Clinique Saint-Augustin, Bordeaux, France

**Keywords:** Non-muscle invasive bladder cancer, Meningeal carcinomatosis, Complete response, Chemotherapy

## Abstract

**Background:**

Non-muscle invasive bladder cancer (NMIBC) is usually treated with local therapy including transurethral resection of the bladder tumor and intravesical therapy depending on the stage of the tumor. NMIBC is a rarely a metastatic diseases with lymph node invasion in less of 10%. In the other hand meningeal carcinomatosis is a rare location for metastases with extremely poor outcomes. We described a case report of a patient presenting a metastatic disease to bones and meninges, several years after the treatment of NMIBC, which had been in complete response (CR) for 4 years after chemotherapy treatment.

**Case presentation:**

A 63-years old men was treated by TURBT in 2008 for a high grade NMIBC, pT1b. Three years later he presented an acute binocular diplopy with right trochlear nerve paralysis, and labial hypoesthesia. Brain scan and MRI were performed finding a clivus infiltration and a pachymeningitis. A vertebral biopsy was performed finding an invasive carcinoma, CK7+/CK20+, TTF1-, PSA-, Thyroglobulin- and GATA3+. The metastatic event was in relation to the high grade NMIBC treated 3 years previously. Palliative chemotherapy was started with cisplatin gemcitabine. After 6 cycles and to date, 4 years later, the patient is therefore considered in complete response.

**Conclusion:**

Metastasis in non-muscle invasive urothelial carcinoma is rare. Meningeal carcinomatosis outcome is poor, usually appearing in widely metastatic and progressive cancers but also because most systemic agents fail to pass the blood-brain barrier and penetrate into the cerebrospinal fluid. We described an unexpected response with complete response after chemotherapy for meningeal carcinomatosis of non muscle invasive urothelial carcinoma.

## Background

Bladder cancers are the eighth most common cancer in Europe, with an incidence of 19.5/100000/year and a mortality rate of 7.9/100000/year. The most frequent histological subtype is urothelial carcinoma. Seventy percent of the cases are non-muscle invasive bladder cancer (NMIBC) [1].

Usually the diagnosis and treatment of NMIBC consists in a transurethral resection of the bladder tumor (TURBT). A second TURBT is recommended and intravesical therapy may be carried out after complete resection depending on the stage of the tumour.

Unfortunately, despite treatment, 31 to 78% of patients experience a tumor recurrence within 5 years. In 5 to 20% of the cases it progresses to a muscle-invasive bladder cancer [2].

A recent study showed that 10% of the NMIBC patients who underwent a radical cystectomy had a lymph node invasion (9% for T1, 12% for Tis, 4.5% for Ta) [3].

Otherwise meningeal carcinomatosis (MC) is a rare location for metastases from a solid tumor and is correlated with an extremely poor outcome, with a median survival of 4 to 6 weeks [4].

We report here on an original case of a patient presenting a metastatic disease to bones and meninges, several years after the treatment of NMIBC, which had been in partial response (PR) with prolonged disease-free-survival for 4 years after a single line of chemotherapy consisting of Cisplatine and Gemcitabine.

## Case presentation

In July 2011, a 63-year old male patient complained of an acute binocular diplopy with right trochlear nerve paralysis, and labial hypoesthesia.

Previous medical history included a high grade NMIBC, pT1b, treated in 2008 by TURBT and intravesical BCG therapeutic therapy. The patient presented no signs of local recurrence. He had ceased smoking after 60 Pack-Years.

Brain scan and MRI were performed finding a clivus infiltration and a pachymeningitis.

There were no infectious or autoimmune diseases, three spinal taps showed no bacteria, abnormal cells, or hyperproteinorachy, and the tumor markers (AFP, PSA, NSE, ACE, CA19.9, thyroglobulin, calcitonin) were negative.

The patient underwent a whole body CT-scan revealing blastic lesions mostly in the spinal and femoral regions. A PET-scan was then performed, showing a diffuse bone marrow hypermetabolism but no primary tumor site. Additional work-up included a cystoscopy, a prostate echography, a thyroid echography and a gastrointestinal endoscopy. All were negative.

Finally, a vertebral biopsy was performed finding an invasive carcinoma, CK7+/CK20+, TTF1-, PSA-, Thyroglobulin- and GATA3 + .

Based on the patient’s medical history and the immunohistochemical analogy we hypothesized that the metastatic event was in relation to the high grade NMIBC treated 3 years previously.

Palliative chemotherapy was then initiated with 6 planned cycles combining gemcitabine-cisplatine, with a mid-term assessment.

Chemotherapy was safely administered with with no side effects other than Grade 1 nausea and diarrhea for 4 days at each cycle. Due to the cumulative toxicity of the Gemcitabine, the last injection was not given because of a Grade 2 thrombocytopenia.

After 3 cycles, the binocular diplopy disappeared, the brain MRI found a decrease in the meningeal rising, and the PET-scan reported a decrease in bone metabolism but continued persistence of the heterogeneity of clivus and bones.

**Fig. 1:**
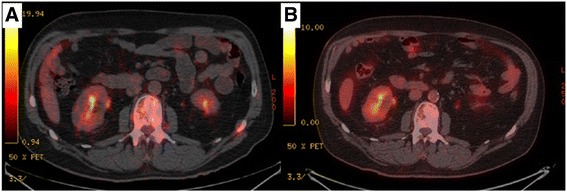
PET-scan before (**a**) and after (**b**) treatment showing a decrease in bone hypermetabolism

**Fig. 2:**
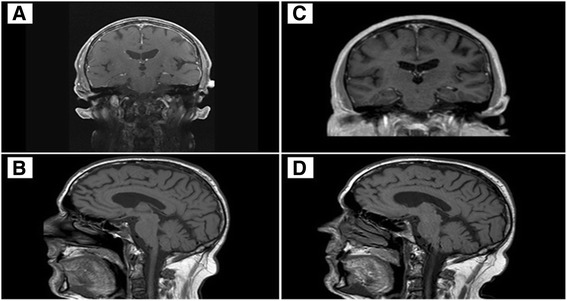
Brain MRI before (**a**, **b**) and after (**c**, **d**) treatment, showing absence of the pachymeningitis and a refilling of the clivus

**Fig. 3:**
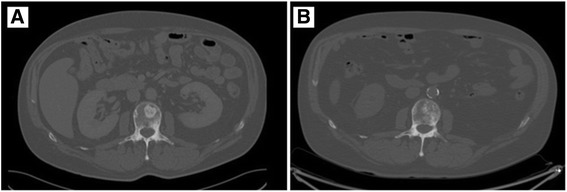
CT-scan before (**a**) and after (**b**) treatment showing a persistence of bone infiltration

After 6 cycles and to date, 4 years after the last administration of chemotherapy, there is no further PET-FDG metabolism in brain or bones, but persistence of heterogeneity (Figs. 1, 2 and 3) on MRI. The patient is therefore considered to be in complete response (CR).

Clinically, he has no other neurological symptom but a restricted labial hypoesthesia.

The interest of complementary cerebral radiotherapy was challenged in a multidisciplinary meeting. However, considering the complete response and the expected long term toxicity of a whole brain irradiation, the indication was not retained.

The patient still has his bladder. The last cystoscopy was performed in September 2015 and was normal.

## Discussion and conclusion

This undifferentiated carcinoma can be linked to the NMBIC. However, CK7 and CK20 positivity is more likely to be correlated with urothelial, pancreatic, ovarian and cholangiocarcinoma, and GATA 3 is correlated with breast, cutaneous and urothelial cancers [5].

Metastasis in non-muscle invasive urothelial carcinoma is rare, and in this case the patient’s NMIBC has never recurred. Matthew and al. [6] reviewed cases of more than 1000 patients treated by radical cystectomy for bladder cancer, regardless of the stage, and identified 9 patients with superficial urothelial carcinoma and distant metastatic disease (including 3 patients with stage pTa disease).

The location of the metastases is atypical in this case since urothelial carcinoma is more likely to metastasize in the liver (47%), lung (45%) or bones (32%) but rarely in the meninges (5%) [7, 8].

Visceral metastases are correlated with a poor outcome in bladder cancers. Overall survival (OS) decreases from 18 months in inoperable locally advanced bladder cancers, to 8.5 months in the case of visceral metastases, and even to 38 days in the case of MC [9, 10].

MC outcome is poor, usually appearing in widely metastatic and progressive cancers (70%) but also because most systemic agents fail to pass the blood-brain barrier and penetrate into the cerebrospinal fluid [11].

The first-line therapy for metastatic bladder cancer is based on a doublet of chemotherapy with platinum.

Historically it was MVAC (methotrexate, vinblastine, doxorubicine, cisplatine) with an overall response rate (ORR) of 72**%** and a median survival lasting more than a year [12] However, treatment with MVAC is associated with substantial toxicity. There was 82% Grade 3-4 neutropenia, and 25% nadir sepsis, as well as significant mucositis (13% Grade 3 or 4).

A new trial, testing Gemcitabine-Cisplatine (GC) versus MVAC, was therefore designed showing a similar survival advantage: OS was 13.8 months vs 14.8 months, and ORR was 49% vs 46% in the GC and MVAC arm respectively. Less toxicity, especially regarding neutropenia (Grade 3-4: 82 vs 71%) and mucositis (Grade 3-4: 22% vs 1%), was noted in the GC arm [13].

The Gemcitabine-Cisplatine combination is therefore now considered to be the standard care.

We report a PR with prolonged disease-free-survival, which is spectacular given the location of metastases, especially the meningeal carcinomatosis. Von der Maase and al. [13] showed that we could expect 12% of complete response in locally advanced or metastatic urothelial carcinoma with Gemcitabine-Cisplatine treatment, not taking into account the location of the metastases, and the median time to relapse after a CR is unknown but probably far less than 4 years.

In summary, in this case we observed an unexpected response, lasting in time, after only 6 cycles of Gemcitabine-Cisplatine, in a rare metastatic non-muscle invasive urothelial carcinoma, with a poor outcome given the location of the metastases and particularly with a meningeal carcinomatosis.

## Abbreviations

CR: Complete response
GC: Gemcitabine-Cisplatine
MC: Meningeal carcinomatosis
MRI: Magnetic resonance imaging
MVAC: Methotrexate, vinblastine, doxorubicine, cisplatine
NMIBC: Non-muscle invasive bladder cancer
ORR: Overall response rate
OS: Overall survival
PET-FDG: Positron Emission Tomography 18-Fluoro-deoxyglucose
TURBT: Transurethral resection of the bladder tumor
